# Posterior reversible encephalopathy syndrome mimicking subacute ischemic stroke: a case report^☆^

**DOI:** 10.1016/j.radcr.2022.06.013

**Published:** 2022-06-21

**Authors:** Soichiro Yamaguchi, Hideki Endo, Yuma Hiratsuka, Hirohiko Nakamura

**Affiliations:** Department of Neurosurgery, Nakamura Memorial Hospital, South 1, West 14, Chuo-ku, Sapporo, Hokkaido 060-8570, Japan

**Keywords:** Cerebral infarction, Hypertension, Ischemic stroke, Magnetic resonance imaging, Posterior reversible encephalopathy syndrome, Vasogenic edema

## Abstract

Posterior reversible encephalopathy syndrome, an acute onset neurological syndrome, is among the conditions that must be differentiated from stroke. Herein, we report a rare case of posterior reversible encephalopathy syndrome mimicking subacute ischemic stroke. A 68-year-old man was transferred by ambulance to our hospital because of visual disturbance. He showed left homonymous hemianopsia. Magnetic resonance imaging (diffusion-weighted imaging and fluid-attenuated inversion recovery imaging) revealed high signal intensity in the right occipital lobe. We suspected subacute cerebral infarction. After admission, he developed cortical blindness and increased blood pressure. Fluid-attenuated inversion recovery imaging revealed high signal intensity and elevated apparent diffusion coefficient values in the bilateral occipital lobes. We diagnosed the patient with posterior reversible encephalopathy syndrome. Antihypertensive treatment improved his clinical symptoms. Careful imaging assessment, including of changes over time, is important for diagnosing posterior reversible encephalopathy syndrome.

## Introduction

Posterior reversible encephalopathy syndrome (PRES) typically presents with neurological symptoms, such as encephalopathy due to reversible subcortical vasogenic edema of the bilateral occipital lobes [1], [2], [3]. Although relatively rare, as an acute onset neurological disorder, PRES is among the conditions that must be differentiated from stroke. Herein, we report a rare case of PRES mimicking subacute ischemic stroke.

## Case report

**Fig. 1:**
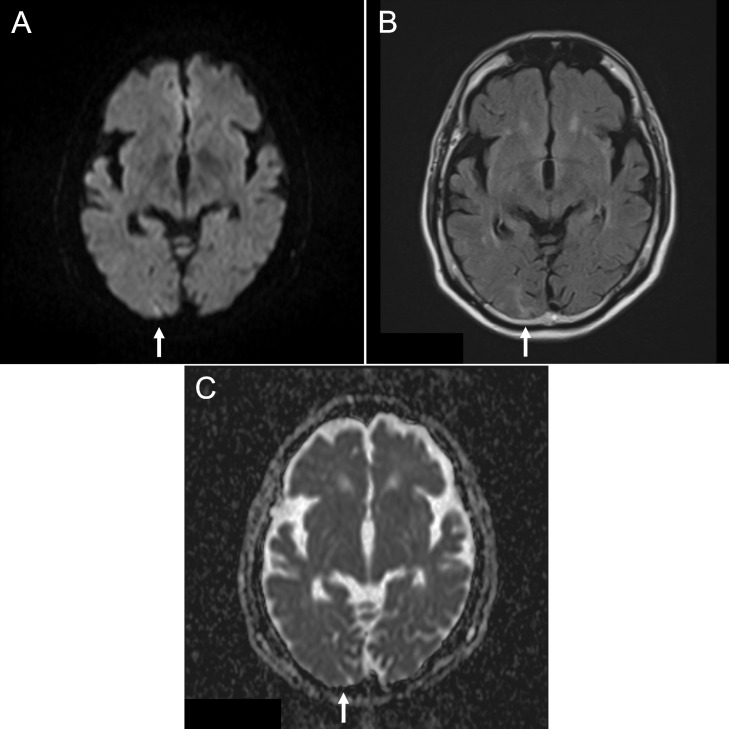
Diffusion-weighted imaging (A) and fluid-attenuated inversion recovery imaging (B) on admission revealed relatively high signal intensity in the right occipital lobe, with no obvious change in apparent diffusion coefficient values (C) (arrows).

A 68-year-old man visited an ophthalmology clinic with dizziness and trouble focusing his eyes since the morning; he had also been experiencing headaches for 2 days. He had a medical history of hypertension, which was well controlled with angiotensin receptor blockers. The patient was transferred by ambulance to our hospital with suspected brain disease. On arrival, he showed left homonymous hemianopsia and his blood pressure was 146/99 mm Hg. Magnetic resonance imaging (MRI; diffusion-weighted imaging [DWI] and fluid-attenuated inversion recovery [FLAIR] imaging) revealed an area of relatively high signal intensity in the right occipital lobe (Figs. 1A and B). No obvious changes were apparent on apparent diffusion coefficient (ADC) maps (Fig. 1C). Magnetic resonance angiography did not reveal steno-occlusive lesions. We suspected subacute cerebral infarct and started heparin treatment after hospitalization.

**Fig. 2:**
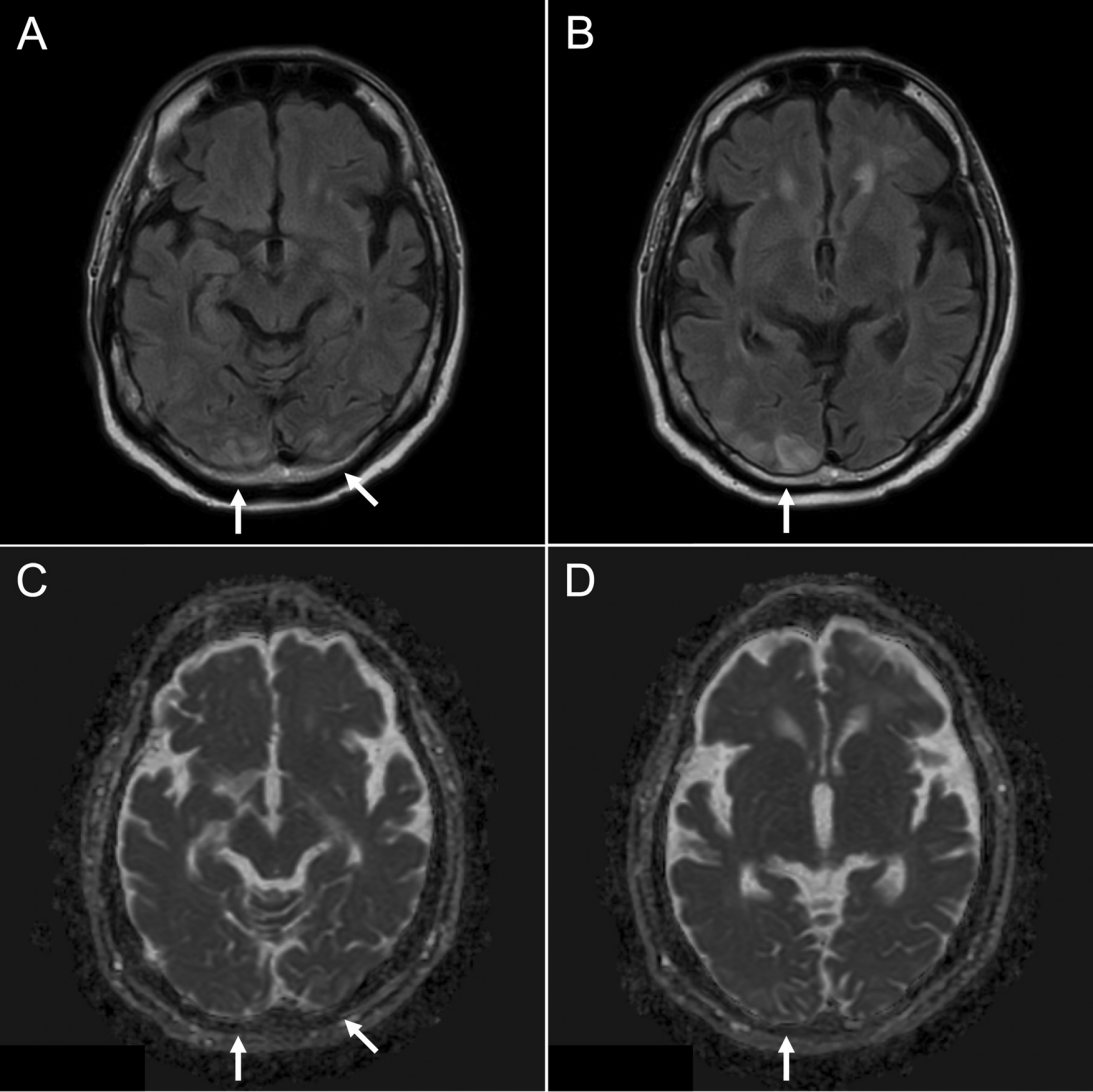
Fluid-attenuated inversion recovery imaging (A, B) at the time of cortical blindness revealed high signal intensity, along with elevated apparent diffusion coefficient values (C, D) in the bilateral occipital lobes (arrows).

The patient complained of deteriorating visual acuity in both eyes the next morning, and his blood pressure had increased to 180/119 mm Hg. FLAIR imaging revealed areas of high signal intensity extending into the bilateral occipital lobes, and ADC values were elevated (Fig. 2). We diagnosed PRES due to severe hypertension and immediately began antihypertensive treatment with nicardipine. We also added an oral calcium channel blocker. His cortical blindness improved, with only mild residual left visual field impairment. He developed tonic-clonic seizures on the third night, which were treated with diazepam and fosphenytoin. The clinical symptoms subsequently improved and the patient was discharged home 2 weeks later.

## Discussion

Herein, we presented a rare case of PRES mimicking subacute ischemic stroke. PRES is a neurological syndrome first reported by Hinchey et al [4] in 1996; its antecedents, pathogenesis, symptoms, imaging findings, and clinical course have been well characterized. At present, there are no clear diagnostic criteria for PRES, although the following have been proposed: (1) acute clinical presentation; (2) presence of known risk factors; (3) reversibility of clinical and/or radiological findings; (4) exclusion of other possible causes of encephalopathy or vasogenic edema; (5) high signal intensity on FLAIR images consistent with PRES-specific imaging patterns; and (6) vasogenic edema, as demonstrated by DWI and ADC values [5]. Our patient presented with typical clinical symptoms (such as visual disturbance, epileptic seizures) and imaging findings of PRES after the second hospital day (Fig. 2).

Imaging findings, and especially MRI findings, is key for differential diagnosis. Since PRES is associated with vasogenic edema, the lesions show high signal intensity on FLAIR images, as well as elevated ADC values, and are typically bilateral [5]. On the other hand, acute ischemic stroke (for which differential diagnosis is required in neurological emergencies) is associated with cytotoxic edema, which has high signal intensity on DWI and lower ADC values. However, subacute ischemic stroke may show high signal intensity on DWI and FLAIR images, and ADC values may be unchanged [6,7,8]. The initial images in the present case showed a pattern of high signal intensity characteristic of subacute ischemic stroke (Fig. 1). Also, the lesion was unilateral and too small to detect any change in the ADC maps, which may have contributed to the failure to diagnose PRES initially. However, the MRI findings at the time of cortical blindness were consistent with PRES (Fig. 2). We also tracked changes in MRI findings over the course of the acute phase; to the best of our knowledge, this was not done in any previous studies.

For successful management of PRES, early diagnosis and treatment initiation are important. Hyperglycemia and failure to control causative factors in a timely manner have been reported as independent poor prognostic factors [1]. Moreover, extensive vasogenic edema, hemorrhage, and restricted diffusion on initial imaging were associated with worse clinical outcomes of PRES patients [9]. Although the initial MRI (DWI) of our patient showed high signal intensity, symptomatic improvement was achieved with antihypertensive treatment started immediately after diagnosis. These findings indicate the importance of early therapeutic intervention to avoid a poor clinical prognosis.

## Conclusions

Herein, we described a rare case of PRES mimicking subacute ischemic stroke. MRI findings are key for diagnosing PRES, but may be insufficient for diagnosis in the acute phase. Careful imaging assessment, including of changes over time, is important for the diagnosis and early treatment of PRES.
